# Entinostat augments NK cell functions via epigenetic upregulation of IFIT1-STING-STAT4 pathway

**DOI:** 10.18632/oncotarget.27546

**Published:** 2020-05-19

**Authors:** John M. Idso, Shunhua Lao, Nathan J. Schloemer, Jeffrey Knipstein, Robert Burns, Monica S. Thakar, Subramaniam Malarkannan

**Affiliations:** ^1^Laboratory of Molecular Immunology and Immunotherapy, Blood Research Institute, Versiti, Milwaukee, WI, USA; ^2^Division of Pediatric Hematology-Oncology-BMT, Department of Pediatrics, Medical College of Wisconsin, Milwaukee, WI, USA; ^3^Bioinformatics Core, Blood Research Institute, Versiti, Milwaukee, WI, USA; ^4^Divson of Hematology-Oncology, Department of Medicine, Medical College of Wisconsin, Milwaukee, WI, USA; ^5^Department of Microbiology and Immunology, Medical College of Wisconsin, Milwaukee, WI, USA; ^*^Co-senior authors

**Keywords:** NK cells, histone deacetylase inhibitor, Ewing sarcoma, rhabdomyosarcoma, immunotherapy

## Abstract

Histone deacetylase inhibitors (HDACi) are an emerging cancer therapy; however, their effect on natural killer (NK) cell-mediated anti-tumor responses remain unknown. Here, we evaluated the impact of a benzamide HDACi, entinostat, on human primary NK cells as well as tumor cell lines. Entinostat significantly upregulated the expression of NKG2D, an essential NK cell activating receptor. Independently, entinostat augmented the expression of ULBP1, HLA, and MICA/B on both rhabdomyosarcoma and Ewing sarcoma cell lines. Additionally, entinostat increased both cytotoxicity and IFN-γ production in human NK cells following coculture with these tumor cells. Mechanistically, entinostat treatment resulted in increased chromatin accessibility to the promoter region for interferon-induced protein with tetratricopeptide repeats 1 (*IFIT1*) gene and thereby increasing the transcript and protein levels of IFIT1 that augmented the IFIT1-mediated IRF1, STAT4, and STING pathways. Corresponding transcriptome analysis revealed enrichment of *IRF1* and *STAT4* and gene sets responsible for NK cell-mediated IFN-γ production and cytotoxicity, respectively. Our results show a novel mechanism by which entinostat initiates an IFIT1-STING-mediated potentiation of STAT4 via IRF1 to augment NK cell-mediated anti-tumor responses.

## INTRODUCTION

Histone deacetylase inhibitors (HDACi) represent an essential class of antineoplastic medications due to their ability to restore the function of proteins that reverse the growth and progression of a wide array of cancers with relatively minimal toxicities [1, 2]. HDACi is used alone or in combination with other chemotherapeutic agents for a variety of pediatric and adult malignancies, including rhabdomyosarcoma and cutaneous T cell lymphoma [1, 3]. Their emerging clinical potential has led to the evaluation of HDACi for tumor treatment of several cancer types in various trials [4]. Irrespective of this, the molecular mechanism by which HDACi modulate immune responses has yet to be fully understood [1]. Earlier studies primarily focused on the effect of HDACi on tumor cells, while a few evaluate their impact on the immune system [5–12]. Specifically, the effect of HDACi on natural killer (NK) cells has yet to be fully defined.

NK cells are the major lymphocytes of the innate immune system and play an essential role in tumor clearance [13]. NK cells are of particular importance in the context of hematopoietic cell transplant (HCT) as they are the first cell type to recover following engraftment of donor stem cells [14]. One critical concept behind allogeneic HCT in cancer is to replace a senescent recipient’s immune system with one that will recognize and kill malignant cells. NK cell-based immunotherapy is emerging as a safe and effective treatment against pediatric solid tumors such as neuroblastomas and sarcomas [15, 16]. In addition, while these strategies have demonstrated efficacy in hematologic malignancies, neither in its current form has yielded consistently curative effects in solid malignancies [17]. This is attributed to factors associated with the tumor microenvironment that mediate resistance external to the immune cells [18, 19]. Thus, it is necessary to increase the sensitivity of the immune cells to the tumor. Strategies to overcome these tumor-tolerance factors include use of chimeric antigen receptors (CAR), granulocyte-monocyte colony-stimulating factor (GM-CSF), interleukin (IL)-2, IL-15, checkpoint inhibitors, and type 1 interferons [20–23]. While these strategies have shown efficacy, their use is limited due to significant, even lethal, toxicities. However, it is likely that NK cell-based immunotherapy will require strategies to overcome the tumor microenvironment. Thus, a nontoxic method to augment NK cell-based immunotherapy is urgently needed.

We hypothesize that overcoming tumor microenvironment-mediated tolerance in the epigenome of the NK cell can increase tumor clearance. Entinostat (SNDX-275, formerly MS-275) has selective activity against class I histone deacetylases [24, 25]. Previous studies have shown that HDACi may exert their antineoplastic effects through 1) upregulation of immunostimulatory ligands on tumor cells and 2) upregulation of activating receptors on lymphocytes [26–30]. However, the molecular mechanism associated with augmented NK cell function has not been defined. Here, we show that entinostat treatment increased both activating and inhibitory receptors on NK cells. Coculture of NK cells and tumors demonstrated an increase in both cytotoxic degranulation and IFN-γ production in NK cells. Transcriptome analysis revealed the enrichment of pathways associated with NK cell cytotoxicity and interferon-gamma (IFN-γ) production. We identify the enrichment of a gene set associated with transcription factors interferon regulatory factor-1 (*IRF1)* and signal transducer and activator of transcription 4 (*STAT4)* in NK cells following treatment with entinostat. Chromatin structure analysis using Assay for Transposase Accessible Chromatin (ATAC) sequencing indicates an area of open chromatin at the transcription start site for interferon-induced protein with tetratricopeptide repeats 1 *(IFIT1)*. Analyses of the transcriptome were consistent with the upregulation of *IFIT1* expression and subsequent NK cell activation via IRF1 and STAT4. Collectively, our results provide a novel mechanism of action (MOA) of entinostat-regulated NK cell effector functions and identify targets that could help augment NK cell-mediated anti-tumor responses.

## RESULTS

### Entinostat upregulates the expression of activating and inhibitory human NK cell receptors

**Figure 1:**
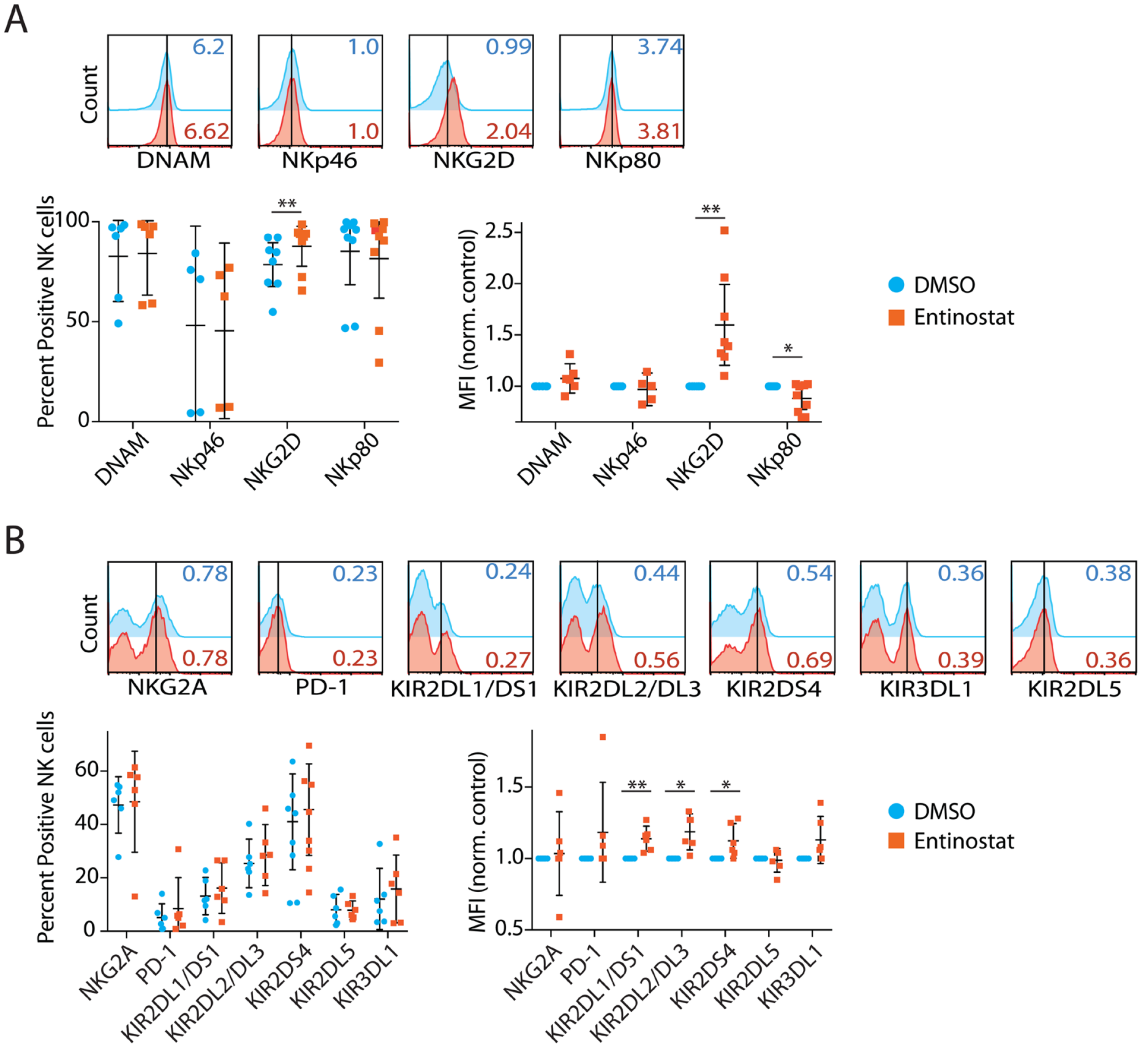
Entinostat upregulates the expression of activating and inhibitory human NK cell receptors. (**A**) Effect of entinostat on NK cell-activating receptors, including DNAM, NKp46, NKG2D, and NKp80. The total percent receptor-positive of the CD3ε^−^CD56^+^ NK cells and their Mean Fluorescent Intensity (MFI) normalized to DMSO control are shown. (**B**) Effect of entinostat on NK cell inhibitory receptors including NKG2A, PD-1, KIR2DL1, KIR2DL2, KIR2DS4, KIR2DL5, and KIR3DL1. Data are shown as percent receptor-positive of the CD3ε^−^CD56^+^ NK cells and their MFI normalized to DMSO control are shown. Data shown in A and B are obtained by treating purified NK cells with or without entinostat from five to seven healthy donors per group. Data presented are the mean ± SD. Statistical significance was calculated using a ratio paired *t-test*. ^*^ *p* < 005; ^**^ *p* < 0.01.

The balance between activating and inhibitory receptors determines whether the NK cell recognition of a target cell results in the activation of effector functions or tolerance [31]. Earlier work has indicated that HDACi enhances the expression of NK cell receptors, including NK Group 2D (NKG2D), a key activation receptor [27]. To determine the effect on the expression of activating and inhibitory receptors, we incubated NK cells with entinostat, a benzamide HDAC inhibitor. We sorted CD56^+^CD3ε^−^ NK cells from the peripheral blood of volunteers, and the purity of the NK cells ranged between 96–99% (Supplementary Figure 1A). First, we tested the effect of entinostat on the viability of the purified NK cells. Incubation of sorted NK cells with entinostat for 24 hours resulted in no cell death (Supplementary Figure 1B). Following this, we analyzed the effect of entinostat on the expression of activating receptors expressed on NK cells. We quantified both the percent positive among NK cells and the mean fluorescent intensity (MFI) to determine the changes at both cell population and receptor density on per cell basis. We tested four NK activating receptors DNAX Accessory Molecule-1 (DNAM-1), Natural Cytotoxicity Triggering Receptor 1 (NCR1, also known as NKp46), NKG2D, and Killer Cell Lectin Like Receptor F1 (KLRD1, also known as NKp80) expression using flow cytometry. Surface expression of NKG2D was increased 12% by percent positive within among NK cells (*p* = 0.006) and 54% by MFI (*p* = 0.003) (Figure 1A). However, NKp80 decreased 13% percent by MFI (*p* = 0.03), but not percent positive cells (*p* = 0.17). DNAM-1 and NKp46 were not significantly changed based on percent positive cells and MFI.

We next tested NK Group 2A (NKG2A), PD-1, and inhibitory isoforms of Killer Cell Immunoglobulin-Like Receptors, including KIR2DL1, KIR2DL2, KIR2DS4, KIR3DL1, and KIR2DL5 (Figure 1B). NK cell inhibitory receptors, such as Programmed Cell Death 1 (PD-1), play an important role in mediating tolerance [32]. Surface expression of KIR2DL1 was increased 16% (*p* = 0.11) and 13% by MFI (*p* = 0.009). Surface expression of KIR2DL2 was increased 11% by percent positive cells (*p* = 0.11) and 18% by MFI (*p* = 0.01). Surface expression of KIR2DS4 was increased 19% by percent positive cells (*p* = 0.07) and 12% by MFI (*p* = 0.04). Expressions of NKG2A, PD-1, KIR2DL5, and KIR3DL1 were not significantly altered by percent positive cells or MFI. In summary, while entinostat significantly increased surface expression of activating NKG2D, it also increased the expression of inhibitory receptors, including KIR2DL1, KIR2DL2, and KIR2DS4.

### Entinostat upregulates activating ligands on human tumor cells

**Figure 2:**
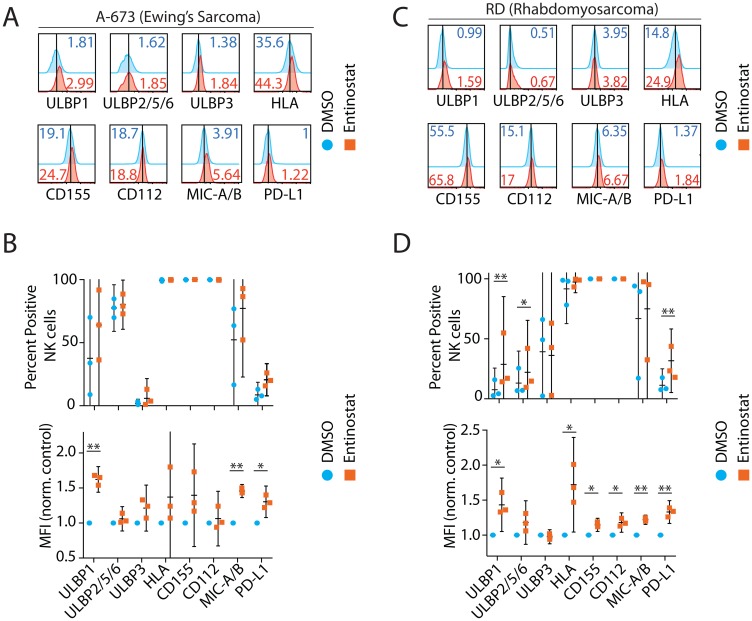
Entinostat upregulates activating ligands on human tumor cells. Ligands selected based on their known interaction with NK receptors include ULBP1, ULBP2,5,6, ULBP3, HLA, CD155, CD112, MICA/B, and PD-L1. Representative flow cytometry histograms of NK receptor ligands for (**A**) A-673 (Ewing Sarcoma) and (**B**) Effect of entinostat on A-673 as measured by percent positive of the CD3ε^−^CD56^+^ NK cells and by MFI normalized to DMSO control. (**C**) RD (Rhabdomyosarcoma) and (**D**) Effect of entinostat on RD as measured by percent positive CD3ε^−^CD56^+^ NK cells and by MFI normalized to DMSO control. Data shown in (A–D) are obtained by treating purified NK cells with or without entinostat from three healthy donors per group. Data presented are the mean ± SD. Statistical significance was calculated using a ratio paired *t-test*. ^*^ *p* < 005; ^**^ *p* < 0.01.

We next sought to define the role of entinostat on two tumor cell lines, A-673 (Ewing sarcoma, Figure 2A, 2B) and RD (Rhabdomyosarcoma, Figure 2C, 2D). These tumors were selected for their relative prevalence in pediatric patients and prior demonstration of sensitivity to NK cytotoxicity [33]. Following entinostat treatment, we evaluated ligands that are expressed on tumor cells and are known to activate or inhibit NK cell functions. This included activating ligands CD112, CD155, MHC I Chain-related molecules A and B (MICA/B), and UL16-binding proteins (ULBP) [31]. We also analyzed the inhibitory ligands, Human Leukocyte Antigen Class-I (HLA), and Programmed death-ligand 1 (PD-L1). MICA and MICB are recognized by NKG2D, resulting in the activation of NK cells. Following exposure to entinostat, the surface expression of MICA/B in RD cells was increased 28% by percent positive among NK cells (*p* = 0.34) and 22% by MFI (*p* = 0.004) (Figure 2A–2D). Similarly, the ULBP family of ligands are also recognized by NKG2D and play critical to NK cell-mediated anti-tumor activity [34]. Expression of ULBP1 was increased 420% by percent positive among tumor cells (*p* = 0.008) and 43% by MFI (*p* = 0.03). ULBP2/5/6 was increased 69% by percent positive among tumor cells (*p* = 0.04) and 17% by MFI (*p* = 0.13). ULBP3 was not significantly different when analyzed by either percent positive cells or MFI. These results demonstrate that entinostat differentially regulates the expression of activating ligands of NKG2D.

Distinct HLA Class-I molecules serve as the ligand for the NK cell inhibitory receptors, KIR, and NKG2A [31]. Expression of HLA was increased 7% by percent positive among tumor cells (*p* = 0.36) and 71% by MFI (*p* = 0.03) demonstrating a predominantly per cell effect of entinostat. CD155 and CD112 are ligands to the NK receptor DNAM and are upregulated in some tumors [35]. Expression of CD155 and CD112 did not change however increased on per cell basis (15 and 18% by MFI, respectively; *p* = 0.02, *p* = 0.03). PD-L1, the ligand for PD-1, has been studied in a large variety of cancers [36], and its expression increased 290% by percent positive (*p* = 0.006) and 33% by MFI (*p* = 0.01).

In summary, entinostat increased surface expression of MICA/B, ULBP1, ULBP2/5/6, HLA, CD155, CD112, and PD-L1 in RD. In A-673, MICA/B surface expression was increased 73% by percent positive (*p* = 0.21) and 46% by MFI (*p* = 0.001) (Figure 2A–2D). ULBP1 was increased 216% by percent positive (*p* = 0.15) and 62% by MFI (*p* = 0.003). Expression of ULBP2/5/6, ULBP3, HLA, CD155, and CD112 was not significantly changed based on the percent of positive or MFI. However, expression of PD-L1 was increased 151% by percent positive cells (*p* = 0.06) and 30% by MFI (*p* = 0.02). In summary, entinostat increased surface expression of MICA/B, ULBP1, and PD-L1 in A-673. Collectively, entinostat significantly upregulated ligands for both activating and inhibitory NK receptors in both RD and A-673.

### Entinostat augments cytotoxicity and cytokine production in an NK cell-intrinsic manner

**Figure 3:**
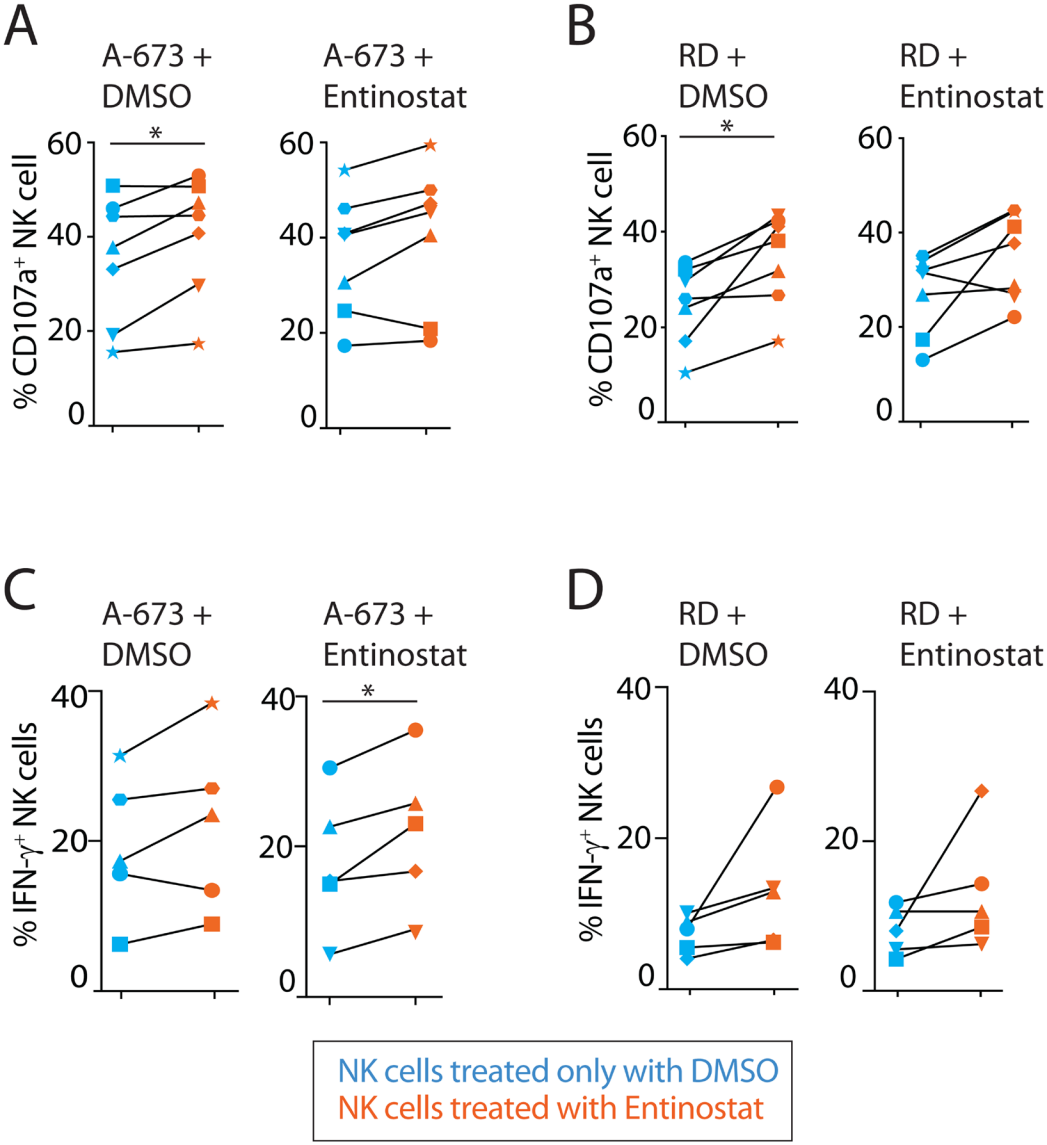
Entinostat augments cytotoxicity and cytokine production in an NK cell-intrinsic manner. (**A**) Effect of entinostat pretreatment on NK degranulation as measured by surface CD107a after 6-hour coculture with an Ewing sarcoma tumor line (A-673). Each scatter plot represents a single tumor pretreatment condition, either DMSO or entinostat. (**B**) Effect of entinostat pretreatment on NK degranulation as measured by surface CD107a after 6-hour coculture with a rhabdomyosarcoma cell line (RD). Each scatter plot represents a single tumor pretreatment condition, either DMSO or entinostat. (**C**) Effect of entinostat pretreatment on intracellular IFN-γ production following 6-hour coculture with A-673. Each scatter plot represents a single tumor pretreatment condition, either DMSO or entinostat. (**D**) Effect of entinostat pretreatment on intracellular IFN-γ production following 6-hour coculture with RD. Each scatter plot represents a single tumor pretreatment condition, either DMSO or entinostat. Data shown in (A–D) are shown as percent positive cells obtained by treating purified NK cells from five to seven healthy donors per group. Blue and orange dots display NK pretreatment with DMSO and entinostat, respectively. Degranulation and IFN-γ production experiments were analyzed using one-way ANOVA with Geisser-Greenhouse correction of the four different experimental comparisons. Post hoc analysis of each pair of experimental comparison was completed using the paired *t-test*. ^*^ *p* < 005; ^**^ *p* < 0.01.

Given entinostat augmented the expression of both activating and inhibitory receptors, it was unclear how this would influence the effector functions of NK cells. We, therefore, sought to define its effect on NK cell-mediated cytotoxicity and IFN-γ production. NK cell-mediated anti-tumor cytotoxicity was measured using flow cytometric analysis to quantify the cell surface expression of the surrogate marker, CD107a (Figure 3A, 3B). CD107a (LAMP1) is localized intracellularly within the cytotoxic granule-containing vesicles of NK cells and upon activation during the exocytosis of granzymes, CD107a molecules are brought to the cell surface [37]. The effect of entinostat on inflammatory cytokine production was assayed by quantifying the intracellular IFN-γ (Figure 3C, 3D). We exposed both NK cells and tumors to entinostat prior to co-culture. Brefeldin-A (Golgi-plug) was added for the last 4 h of activation to prevent the secretion and to assess the intracellular IFN-γ. Pretreatment of both NK cells and tumor with entinostat prior to co-culture resulted in a 47% increase in CD107a-positivity against 14% against A-673 (*p* = 0.003) and RD (*p* = 0.02). These independent pretreatments did not significantly alter the IFN-γ-positive NK cells against RD but with A-673 resulted in an 18% increase (*p* = 0.057). MFI of IFN-γ staining in NK cells resulted in significant change with co-culture with neither RD nor A-673.

To determine whether NK cell or tumor contributed to this effect, we tested them with or without entinostat pretreatment. Entinostat pretreatment of NK cells subsequently co-cultured with untreated tumor cells resulted in a significant increase in NK cell CD107a-positivity (42% vs. RD (*p* = 0.02) and 17% vs A-673 (*p* = 0.03)) (Figure 3A, 3B). The IFN-γ-positive NK cells increased by 62% vs. RD (*p* = 0.057) and no significant change against A-673. IFN-γ MFI of NK cells resulted in no significant change against RD or A-673 (Figure 3C, 3D). Pretreatment of the tumor with entinostat and subsequent co-culture with untreated NK cells resulted in increased CD107a-positivity of NK cells was only 11% against RD (*p* = 0.02) and no significant change when co-cultured with A-673. The IFN-γ-positive NK cells did not significantly change against RD and resulted in a 7% decrease against A-673 (*p* = 0.045). IFN-γ MFI of NK cells resulted in no significant change against RD or A-673 (data not shown). These results suggest that the functional outcome of entinostat is primarily due to its effect on NK cells, as opposed to tumor cells, and that the most important effector function augmented is the NK cell-mediated cytotoxicity.

### Entinostat alters the transcriptome of NK cells to augment their effector functions

**Figure 4:**
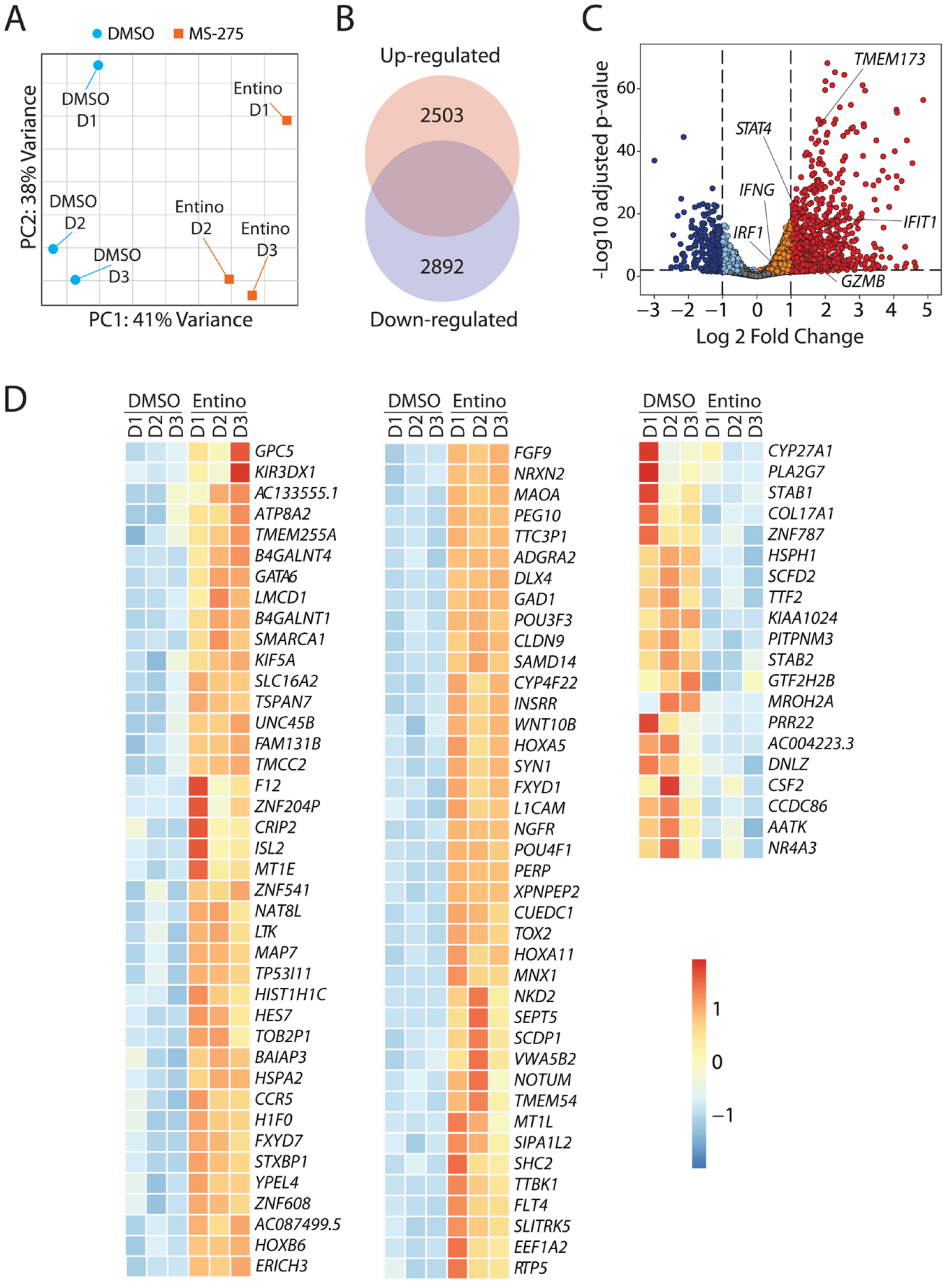
Entinostat alters the transcriptome of NK cells to augment their effector functions. (**A**) Principal Component Analysis (PCA) plot displaying variance attributed to the entinostat-treated and control DMSO-treated groups. (**B**) Venn diagram with the absolute number of genes in the transcriptome that are up-regulated (2503) or down-regulated (2893). (**C**) Volcano plot demonstrating overall changes in the transcriptome with genes of FDR < 0.05 and Log_2_fold-change > 1 in red. The orange/red dots represent genes that are significantly increased, while the aqua/dark blue dots represent genes that are significantly decreased in entinostat-treated compared to the corresponding DMSO-treated human NK cells. (**D**) Heatmap displaying the top 80 and bottom 20 genes by FDR in the transcriptome. Data shown are obtained by treating purified NK cells from three healthy donors per group.

Acetylation of histones is linked to genes that are poised to be transcribed or have been previously transcribed regions of chromatin. Since entinostat affects chromatin structure and gene accessibility, we hypothesized that both would be reflected in the quantitative and qualitative changes of the transcriptome when NK cells are treated with entinostat. To employ an unbiased approach to identify target genes regulated by entinostat, we performed transcriptome-wide RNA sequencing analyses. Purified human NK cells were treated with entinostat for 24 hours, and total mRNA was isolated, transcribed, and sequenced. Unsupervised principal-component analyses (PCA) revealed that the entinostat-treated NK cells possess a transcriptome that is distinct from the non-treated. The percentage variance on the dispersion matrix demonstrates clustering indicating consistency between samples (Figure 4A). We used statistical filtering (*p* < 0.05 DESeq2 Wald-tests, Benjamini–Hochberg correction of treated versus non-treated) to identify genes that were significantly differentially expressed in the entinostat-treated vs. non-treated NK cells (DMSO control).

Initial analyses indicated that entinostat induced a broad transcriptomic alteration in the NK cells. Compared to the DMSO control, entinostat-treated NK cells contained 5395 genes that were differentially expressed (false discovery rate (FDR) < 0.05). Of the genes differentially expressed, 2503 were elevated, and 2892 were decreased (Figure 4A). Overall, the expression of a larger number of genes was augmented in the entinostat-treated NK cell group (Figure 4B and Supplementary Figure 2). After normalizing the level of each transcript in the entinostat-treated to non-treated NK cells, we plotted all the genes using the volcano plots, demonstrating the global change in the transcriptomic profile (Figure 4C). The orange/red dots represent genes that are significantly increased, while the aqua/ dark blue dots represent genes that are significantly decreased in entinostat-treated NK cells compared to the untreated counterparts. Several key transcripts, including *IFIT1*, *TMEM173* (STING), *STAT4*, *IRF1*, *IFNG*, and *GZMB,* were present at a higher level in entinostat treated compared to non-treated NK cells (Figure 4C).

Incubation of NK cells with entinostat resulted in a broad spectrum of alterations in gene expression. Based on the RNA-seq data, we identified the top 80 genes whose transcript levels were augmented and bottom 20 transcripts that were down-regulated following entinostat treatment (Figure 4D). These transcripts represented a wide variety of cellular functions. This included transcription factors (*GATA6*, *CRIP2*, *TOX2*, *HES7*, *POU3F3*), Chromatin remodeling (*SMARCA1*, *LMCD1*), molecules involved in glycosylation/acetylation (*GPC5*, *B4GALNT4*, *B4GALNT1*, *NAT8L*), metabolism (*CYP4F22*, *INSRR*, *GAD1*), and genes involved in cell division and differentiation (*KIF5A*, *TSPAN7*, *LTK*, *MAP7*, *NKD2*, *YPEL4*, *HOXB6*, *HOXA5*, *HOXA11*, *L1CAM*, *NGFR*). The transcripts that are downregulated included Transcription termination factor 2 G (*TTF2*), Stablin1/2 (*STAB1*/*2*), Phospholipase A2 G-VII (*PLA2G7*), and Granulocyte-macrophage colony-stimulating factor (*CSF2*). Collectively, these results indicate that inhibition of histone deacetylases by entinostat causes considerable alterations in the expression of genes that are involved in growth arrest, differentiation, and cell metabolism.

### Entinostat enriches genes governing effector functions of NK cells

**Figure 5:**
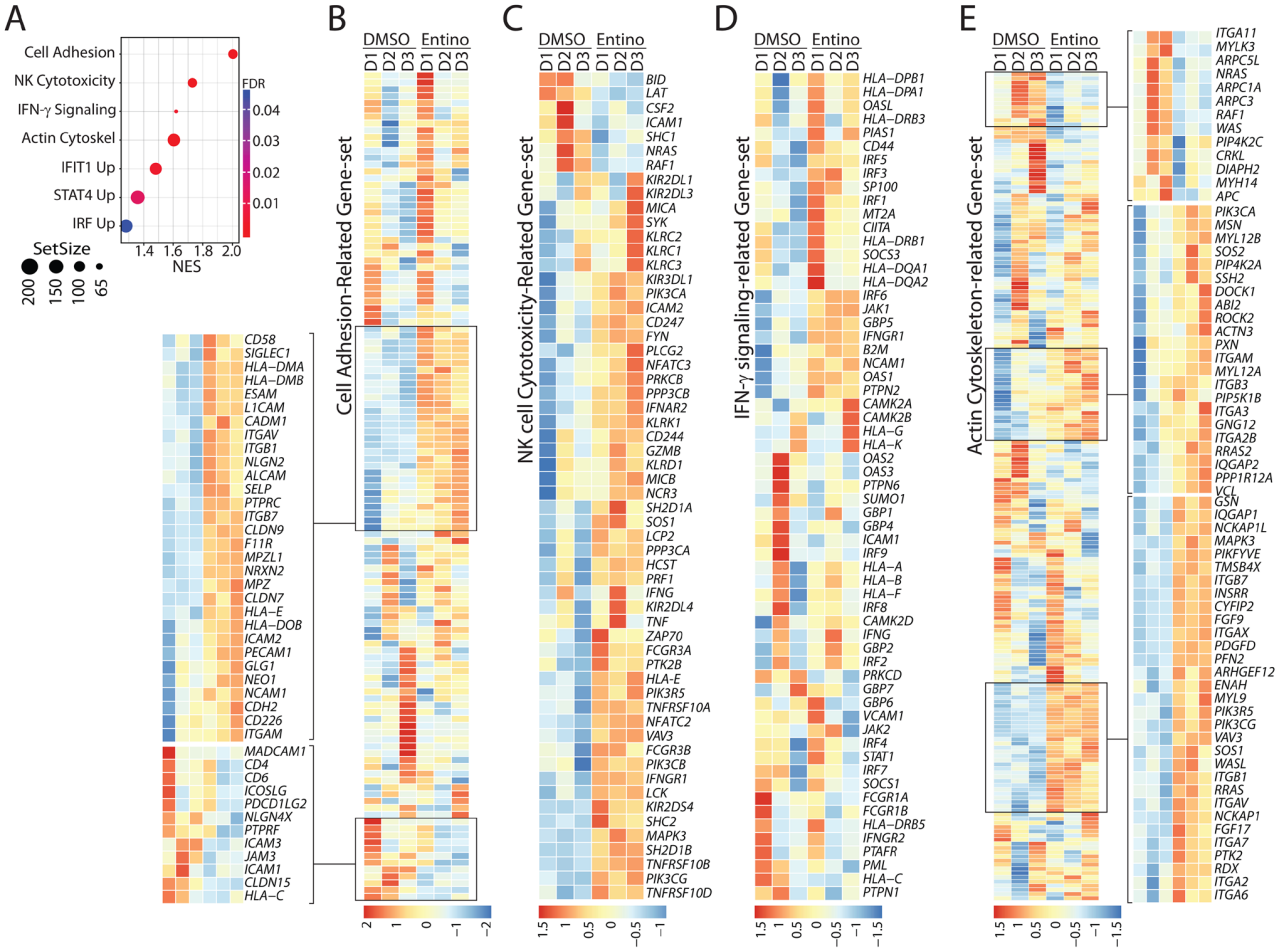
Exposure to entinostat increases the expression of gene-sets involved in the effector functions of NK cells. (**A**) Gene Set Enrichment Analysis (GSEA) results displayed according to the pathway with FDR, normalized enrichment score, and size of the gene set. Normalized enrichment score (NES) analyses revealed enrichment of gene-sets involved in cell adhesion, NK cell-mediated cellular cytotoxicity, IFN-γ-mediated signaling, and actin-cytoskeletal remodeling. Enrichment score were normalized to mean enrichment of random samples of the same size within the RNA-seq data. (**B**) Gene-set associated with NK cell adhesion are shown. Heatmap displaying genes with FDR < 0.05 associated with cell adhesion. (**C**) Heatmap displaying genes with FDR < 0.05 of NK Cell Cytotoxicity gene set. Gene-set associated with the cytotoxic functions of NK cells are shown. (**D**) Heatmap displaying genes with FDR < 0.05 of IFN-γ-mediated signaling-related gene set. Gene-set associated with IFN-γ-mediated signaling are shown. (**E**) Heatmap displaying genes with FDR < 0.05 of gene-set related to cytoskeletal reorganization. Gene-set associated with the actin-based cytoskeletal reorganization are shown. Data shown are obtained from RNA-Seq analyses of NK cells from three healthy donors with or without entinostat treatment.

To identify the unique pathways that were initiated in NK cells by entinostat-treatment, we utilized the Gene Set Enrichment Analysis (GSEA). Curated gene sets from the Kyoto Encyclopedia of Genes and Genomes (KEGG) including Cell Adhesion Molecules (CAM), Natural Killer Cell-Mediated Cytotoxicity (NKCMC), Interferon Gamma Signaling (IGS), and Regulation of Actin Cytoskeleton (RAC) from Reactome were identified (Figure 5A) [38]. Normalized enrichment scores (NES) were calculated against mean enrichment scores of random gene-set samples of the same size. CAM gene set consists of 121 genes and was elevated in the treatment group with an NES of 2.01 (FDR = 0.005). Entinostat treatment upregulated NCAM1 (CD56) and CD226 (DNAM-1), indicating its direct effect on NK cell-specific adhesion and activation receptors (Figure 5B). Although the transcript level of DNAM-1 increased, we did not observe any corresponding increase in our flow analyses (Figure 1A). This increase in the transcript levels of Besides, several genes encoding cell adhesion molecules were either upregulated (*CD58*/LFA3, *SIGLEC1*, *ALCAM*, *ICAM2*, *PECAM1*/CD31 and integrins *ITGAV*/αV, *ITGB1*, *ITGB7*, *ITGAM*/αM) or downregulated (MADCAM1, CD4, CD6/Scavenger receptor cysteine-rich, *ICAM3*, *ICAM1*, *JAM3*). Other genes elevated in this pathway by FDR include *L1CAM*, *HLADMB*, *HLADMA*, and *NRXN2* (Figure 5B).

NKCMC gene set includes 118 genes increased in the entinostat-treated group with an NES of 1.72 (FDR = 0.005). (Figure 5C). Since the NK cell-mediated cytotoxicity is vital in clearing solid tumors and given entinostat augments this effector function, we further explored the NKCMC gene set (Figure 5C). Transcripts encoding a number of cell surface receptors including inhibitory receptors were increased They are, *KIR2DL1* (CD158A), *KIR2DL3* (CD158B2), *KLRC1* (NKG2A, CD159A), *KIR3DL1* (NKB1, CD158E), and *KIR2DL4* (CD158D); activating receptors: *KLRC2* (CD159C, NKG2C), *KLRC3* (NKG2E), *KLRK1* (NKG2D, CD314), CD244 (2B4), *KLRD1* (KP43, CD94), *NCR3* (NKp30, CD337), *FCRG3A* (FCG3, CD16A), and *KIR2DS4* (CD158I); and apoptosis-related receptors: *TNFRSF10A* (DR4, CD261, TRAIL-R1), *TNFRSF10B* (DR5, CD262, TRAIL-R2), and *TNFRSF10D* (DcR2, CD264, TRAIL-R4). The increase in the transcripts encoding KIR2DL3, KIR3DL1, NKG2D, and KIR2DS4 were consistent with the increase in their protein expression (Figure 1A and 1B). However, the protein expression level of NKG2A did not show any increase in the flow cytometry analyses following entinostat treatment (Figure 1B). Importantly, the NKCMC gene set also contained an augmented expression of central signaling molecules (*SYK*, *FYN*, *LCK*, *PIK3CA*/PI (3) K-p85α, *PIK3CB*/PI (3) K-p110β, *PIK3CG*/PI (3) K-p110γ, *PLCG2*/PLC-γ2, *SOS1*, *VAV3*, *ZAP70*, *MAPK3*/ERK1). Molecules involved in the cytotoxic functions of NK cells such as *GZMB* (granzyme-B), *PRF1* (perforin), *IFNG* (Interferon-γ), and *TNF* (TNF-α) were also increased following entinostat treatment (Figure 5C). Transcripts encoding effector molecules such as *GZMB*, *PRF1*, *IFNG*, and *TNF* were also increased following treatment with entinostat.

NK cells produce IFN-γ, which can function as an autocrine in promoting the cell-survival effector functions. The Reactome IGS gene set includes 61 genes and increased in the treatment group with an NES of 1.62 (FDR = 0.005) (Figure 5D). As predicted, several genes encoding Major Histocompatibility Complex (MHC) proteins were upregulated through in this pathway following entinostat treatment. This included HLA-DP, HLA-DQ, HLA-G, HLA-K, HLA-A, HLA-B, HLA-F, HLA-DQ, CIITA, B2M, and HLA-DR. Other genes elevated in this pathway by FDR include genes encoding signaling proteins that are downstream of IFN-γ receptor such as JAK1, IRF5, IRF3, IRF1, and IRF6. Actin-based cytoskeletal remodeling and adhesion are essential cellular processes involved in the effector functions of NK cells. The RAC gene set consists of 198 genes and was enriched in the treatment group with an NES of 1.61 (FDR = 0.005). The genes elevated in this pathway by FDR include cytoskeleton-associated proteins such as DOCK1, ROCK2, PXN, IQGAP2, IQGAP1, MYL9, and WASL (Figure 5E). These findings reveal that the increased effector functions of NK cells induced by entinostat are mediated through a complex mechanism than a mere upregulation of NK cell-activating receptors. Collectively, these results demonstrate that pretreatment of NK cells with entinostat potentiates NK cells to a heightened state of activation.

### Epigenetic upregulation of IFIT1 by entinostat

**Figure 6:**
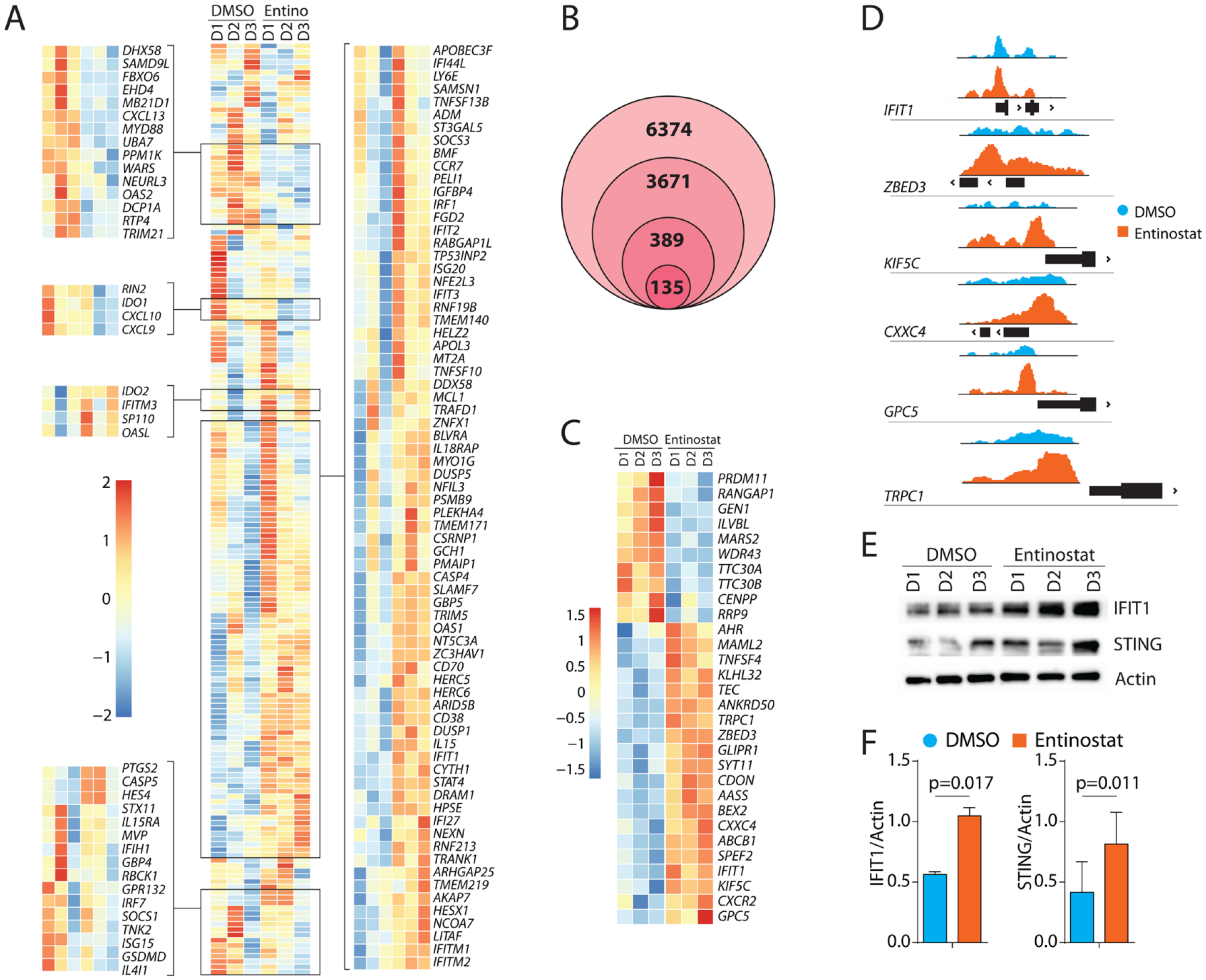
Transcriptomic alterations of IFIT1-associated genes and epigenetic upregulation of IFIT1 by entinostat. (**A**) Gene-expression heatmap of genes associated with IFIT1. Heat map showing genes that are altered in human NK cells following entinostat treatment. Differentially expressed gene-set shown was obtained by comparing RNA-Seq of human NK cells treated with or without entinostat with the previously published gene-set obtained from IFIT1 knockdown in THP1 cells [40]. (**B**) Venn diagram showing filtering of raw (6374) ATAC peaks by protein-coding genes (3671), association with transcription start site (389), and FDR < 0.05 in RNAseq data (135). (**C**) Gene-expression heatmap of the top 30 genes by log_2_fold-change identified as areas of increased chromatin accessibility using ATAC-Seq and had an FDR < 0.05 in the RNAseq. (**D**) ATAC peaks showing the area of increased chromatin accessibility associated with genes with transcriptome log_2_fold-change greater than two using Integrative Genomics Viewer. Y-axis displays transposase accessibility. Peaks are shown about the genomic location of the promoter region (black box) of the respective gene. (**E**) Western blot analyses of IFIT1, STING, and β-actin with or without entinostat treatment. One representative western blot is shown out of three independent experiments. (**F**) Data presented are the mean ± SD three independent western blots. Statistical significance was calculated using a ratio paired *t-test* from three independent experiments.

Next, we sought to identify the transcription factors (TF) that govern the outcome of entinostat treatment. Towards this, we performed GSEA on the TF motifs of gene sets from the Molecular Signatures Database [38, 39]. Notable TF motifs that were significantly elevated included: Interferon-induced proteins with tetratricopeptide repeats-1 (IFIT1), Signal Transducer and Activator of Transcription 4 (STAT4), and Interferon-Regulatory Factor (IRF) (Figure 5A). Among these, the IFIT1-related genes are primarily induced by Type-1 IFNs (IFN-α and IFN-β). IFITs constitute a major gene-set among the Interferon-stimulated genes (ISGs). An earlier study has identified genes associated with an IFIT1 pathway by knocking down its gene using an RNAi screen in a human macrophage-like cell line, THP1 [40]. Using this as a reference gene-set, we identified the transcripts that are altered in human NK cells following entinostat treatment from our RNA-Seq analyses (Figure 6A). This gene set included 185 genes and was enriched with an NES of 1.48 (FDR = 0.005). We found that the expression of multiple IFIT-related genes is augmented including *IFITM3*, *OASL*, *IFIH1*, *IFI44L*, *IFIT2*, *IFIT3*, *OAS1*, *IFIT1*, *IFI27*, *IFITM1*, and *IFITM2*. Notably, IRF1 was also included in this gene set. Moreover, entinostat also augmented the transcription factors that regulate the functions (*NFE2L3*/NRF3, *STAT4*) or development (*NFIL3*/E4BP4) of NK cells. It is important to note that this pathway also revealed that NK cell activation receptors CD70 (TNFSF7) and CD38 (ADP-Ribosyl Cyclase-1) were increased following treatment. Irrespective of the global increase in the increase in the IFIT-related genes, the transcript levels of two IFIT-related genes such as *OAS2*, *IFIH1*, and *ISG15* were reduced (Figure 6A). One of the explanations for this effect of the entinostat could be the differential methylation of the genes. For example, earlier studies have shown that the mRNA levels of OAS2 significantly correlated with number (11 CpG sites) and specific place (CpG site#1) [41].

Since entinostat modulates histone acetylation, we next assessed the chromatin structure. We performed ATAC sequencing on the same samples used for the bulk RNA-Seq. ATAC-seq revealed 6374 areas of differentially accessible chromatin (DAC) in the entinostat-treated group, of which 3671 were associated with protein-coding genes (Figure 6B). Three hundred and eighty-nine areas of DAC were less than 1000 bp from a transcription start site. Of these 389 DACs, 135 were associated with genes also differentially expressed in the RNA-Seq analysis Supplementary Figure 3. If entinostat increases NK cell-mediated cytotoxicity through modulating chromatin structure, we reasoned that the primary regulators of this phenotype would be within this list of 135 genes (Figure 6C). Further filtering of these 135 genes using absolute Log_2_fold-change higher than two from the RNA-Seq data reduced the list to six candidate genes (Figure 6D). This included *IFIT1* (Interferon-induced protein with tetratricopeptide repeats-1), *ZBED3* (Zinc Finger BED type-containing-3), *TRPC1* (Transient receptor potential cation channel, subfamily C-1), *KIF5C* (Kinesin Family Member 5C), *CXXC4* (CXXC Finger Protein 4 or IDAX) and *GPC5* (Glypican Proteoglycan 5). Each gene was investigated for its potential involvement in signaling relevant to NK cells. Collectively, the GSEA analyses and the ATAC-Seq identifies induction of IFIT1 as one of the potential mechanistic links between entinostat and the increase in NK cell-mediated effector functions. Interferon-induced IFIT1 is primarily studied for its role in antiviral activity in non-immune cells [42]. Previous studies of IFIT1 describe its role in potentiating the STING/TBK1 complex [43]. Our results confirm this potentiation with *TMEM173* (STING) (FDR < 0.05) and *TBK1* (FDR < 0.05).

To further validate its role in regulating IFIT1 expression, we incubated purified human NK cells with entinostat for 24 hours and analyzed the cell lysate for the changes in protein expression. We found a significant increase in the protein quantity of IFIT1 and its downstream effector STING (Figure 6E, 6F). For DAC to result in increased gene expression, required transcription factors must be present. Transcription factors for IFIT1 include AML1a, ATF6, GATA-1, IRF9, POU3F2, NFIL3, and BCL6. Of these, *IRF9* had a high number of fragments per kilobase million (FPKM) (mean = 174; Standard deviation = 21) as calculated by the FPKM function in DESeq2. Treatment of NK cells with entinostat also augmented the transcript levels of BCL6 (Figure 4D) and NFIL3 (E4BP4) (Figure 6A). Thus, it is possible that IFIT1 expression is regulated either by direct or indirect action of entinostat.

### Epigenetic upregulation of STAT4 and IRF1/6 by entinostat

**Figure 7:**
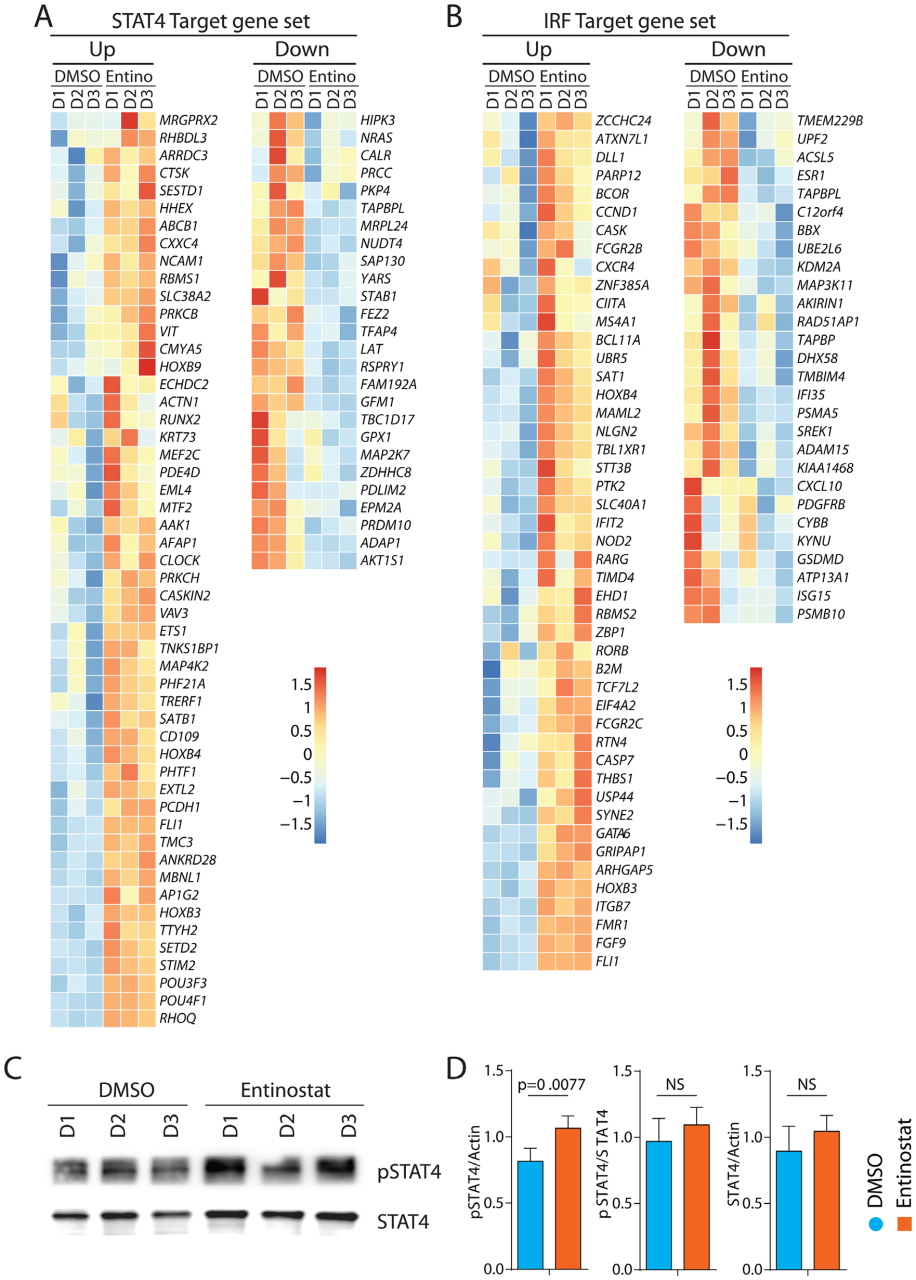
Transcriptomic alterations of target genes of STAT4 and IRF1 by entinostat. (**A**) Heatmap of target genes STAT4. Heatmap displaying genes with FDR < 0.05 of genes targeted by STST4. (**B**) Heatmap of target genes IRF1. Heatmap displaying genes with FDR < 0.05 of genes targeted by IRF1. (**C**) Western blot analyses of pSTAT4 and STAT4 with or without entinostat treatment. One representative western blot is shown out of three independent experiments. (**D**) Specific activation of STAT4 was calculated by quantifying the ratio between β-actin (Figure 6) and phospho-STAT4, phospho-STAT4 and total STAT4, and total STAT4 and β-actin. Western blot analyses shown in Figures 6 and 7 were done at the same time in the same gel. Data presented are the mean ± SD. Statistical significance was calculated using a ratio paired *t-test* from three independent experiments.

In addition to IFIT1-related genes, we found that target genes of STAT4 and IRF1/6 were significantly poised following entinostat treatment. The STAT4 target gene set consists of 266 genes and was enriched with an NES of 1.36 (FDR = 0.009). The top 10 genes elevated in this pathway by FDR include *NCAM1* (CD56), *CD109* (TGF-β1 co-receptor), *CLOCK* (BHLHE8), *RUNX2* (CBF-α1), *SATB1*, *POU4F1*, *PCDH1*, *ABCB1*, *HOXB3*, and *PRKCB* (Figure 7A). IRF1, IRF5, and IRF7 can induce the expression of IFIT and IFITM proteins in the absence of viral infections. The IRF target set consists of 242 genes and was enriched with an NES of 1.28 (FDR = 0.05). The top 10 genes elevated in this pathway by FDR include *CXCR4*, *CIITA*, *SAT1, IFIT2*, *B2M*, *ITGB7*, *FCGR2C*, *FGF9*, *HOXB3*, and *FCGR2B* (Figure 7B). Additionally, the transcripts of the TF genes themselves were significantly elevated including *STAT4* (Log_2_FC = 1.2), *IRF1* (Log_2_FC = 0.6), and *IRF6* (Log_2_FC = 1.7). To confirm the increased activation of STAT4 at a protein level, purified NK cells were incubated with entinostat for 24 hours, and their lysates were analyzed by Western blot. Membranes were probed with antibodies specific for phosphorylated and total STAT4. Our data show an increase in total STAT4 protein (Figure 7C, 7D). TF gene set enrichment coupled with significantly elevated TF transcripts strongly suggests a role for STAT4, IRF1, and IRF6 downstream of entinostat-mediated transcriptomic alteration in NK cells. Consistent with the co-culture cytotoxicity data, these entinostat-induced transcriptomic changes were marked by pathways associated with NK cell activation and function. Based on these results, we conclude that STAT4, IRF1, and IRF6 may be the key mediators of entinostat-induced NK cell activation.

## DISCUSSION

NK cell-based immunotherapy is a promising therapeutic option for high-risk solid tumors [44]. However, NK cell immunotherapy in its current state has limitations, including decreased effector activity in the suppressive tumor microenvironment and lack of persistence in the patient body. HDACi have proven clinical efficacy in cancer therapy [1] and have been shown to augment the anti-tumor functions of NK cells [28, 45]. However, the mechanism of action of HDACi in effector lymphocytes remains elusive. In this study, we evaluated entinostat as an agent to pharmacologically activate human primary NK cells. Entinostat is a Class I HDAC inhibitor and ideal for investigating due to its specificity and favorable safety profile in both Phase I and Phase II trials [46]. We identify that the treatment of NK cells with entinostat initiate an IFIT1-STING-STAT4 innate pathway. The existence of this pathway in NK cells provides novel insights into the mechanism of action of entinostat.

Effector functions of NK cells are coordinated by activating and inhibitory receptors. NKG2D is a primary activating receptor expressed in a majority of human NK cells. NKG2D recognizes ‘stress-induced’ ligands expressed on tumor cells that include MIC-A, MIC-B, and ULBPs. Therefore, we analyzed changes in activating and inhibitory receptors on NK cells and ligands on tumor cells following entinostat treatment. We treated primary NK cells and two tumor cell lines, RD (rhabdomyosarcoma) and A-673 (Ewing sarcoma) with entinostat. Our results identified upregulation of both activating and inhibitory receptors and ligands. This finding was consistent with prior studies showing NKG2D upregulation on NK cells [27] and NK receptor ligands on tumor cells [47, 48]. Entinostat upregulated the expression of NKG2D. Independently, entinostat augmented the expression of ULBP1, HLA, and MICA/B on both rhabdomyosarcoma and Ewing sarcoma cell lines. Next, we co-cultured NK cells and tumor cells and measured degranulation and IFN-γ production following treatment with entinostat. We found a marked increase in NK cell effector functions after treatment with entinostat, consistent with prior literature evaluating NK cell degranulation [28].

To define its mechanism of action, we hypothesized that the mechanism of action of entinostat would involve epigenetic upregulation of one or more genes which would enable the NK cell to alter its transcriptomic profile. We utilized a combination of RNA-seq and ATAC-seq. We found a considerable change in the overall transcriptomic profiles of NK cells treated with entinostat. It is important to note that the concentrations of entinostat used in our study did not cause apoptosis of the human NK cells. This is further validated by the absence of upregulation of apoptosis-related genes in our RNA-seq data. Data from ATAC-seq further validated the upregulation of IFIT1 as the underlying mechanism of the entinostat-induced cytotoxic NK cell phenotype. IFIT1 is an interferon-stimulated gene previously studied primarily for its role in the antiviral response [42]. Additionally, IFIT1 is known to stabilize the STING/TBK1/MAVS complex [43]. Part of this complex, MAVS, has been shown to regulate NK cell maturation [49]. This complex facilitates the increased expression of IRF1 [50], which is an essential mediator of the downstream interferon response. IRF1 is known to bind to the promoter region of STAT4, which is a crucial regulator of IFN-γ production in NK cells [51].

Our results also corroborate previous studies that demonstrated a consistent increase of NK cell receptor ligands on tumor cells [47, 48]. The increase in ligand expression on tumor cells when exposed to HDACi has been well validated in the literature; however, its exact mechanism has yet to be ascertained [28]. If the effect of entinostat on other cell types is also to facilitate increased IFIT1 expression, then a novel mechanism for its observed antitumor effect emerges. In other cells, IFIT1 is involved in the toll-like receptor (TLR) pathway and is thus critical in the antiviral response. During a viral infection, IFIT1 is an essential positive regulator of type I interferon production. It is possible that this mechanism explains the increase in NK-receptor ligand on tumor cells seen in this study and the subsequent increase in sensitivity to killing. Further evidence for this is that IRF1 expression in tumor cells is required for NK-mediated tumor lysis [52].

The results of this study have implications both for NK cell biology and clinical medicine. This is the first time a role for IFIT1-mediated regulation of effector functions has been described in NK cells. IFIT1 has previously been described as a vital signal mediator exclusive to type I interferon signaling. Our model suggests a role for IFIT1 in activating NK cells via STAT4. Previously, HDACi has been understood to exert its antineoplastic effects via ligand upregulation on tumor cells. Our results confirm this effect but also suggest an additional role inducing an anti-tumor phenotype in NK cells.

These results have potential implications for NK cell-mediated immunotherapy in the clinic. Historically, methods to activate NK cells *in vivo* have been effective, but have also induced toxic side effects in patients [53]. Furthermore, while approaches such as cytokine activation can assist in the augmentation of immunotherapy responses, its effect is limited to the cellular side of this treatment. Entinostat is an FDA-approved medication with tolerable side effects [51]. Our results confirm its effectiveness as both an inducer of NK effector functions, but also as an approach to make solid tumors more visible to the immune system by increasing tumor antigen expression. This dual mechanism should be explored as a potential treatment for solid tumors in the development of prospective clinical trials.

## MATERIALS AND METHODS

### Primary NK cell isolation

De-identified healthy human peripheral blood samples were obtained from volunteer donors from the Versiti (Milwaukee, WI). Lymphocytes were enriched using Lymphoprep (STEMCELL Technologies, Vancouver, Canada) and NK cells were isolated to the purification of ~95% using EasySep (STEMCELL Technologies, Vancouver, Canada). After isolation, NK cells were cultured in RPMI 1640 with 10% FBS, 1% penicillin and streptomycin, 1% sodium pyruvate, and 100 IU/mL IL-2. NK cells were immediately separated into 1% DMSO vehicle control and 1% DMSO with 0.5 μM entinostat. After 24 hours of incubation, NK cells were either analyzed directly using flow cytometry or used for degranulation assay.

### Tumor cell lines

The A-673 (Ewing sarcoma) and RD (rhabdomyosarcoma) tumor cell lines were purchased from ATCC (Rockville, MD). A-673 and RD were maintained in tumor media of Dulbecco’s Modified Eagle’s Medium containing 10% FBS. Tumor cells were suspended using 1 mM EDTA in PBS. They were then resuspended in tumor media and separated into two groups, 1% DMSO vehicle control and 1% DMSO with 0.5 μM entinostat. After 24 hours of incubation, cells were resuspended using 1 mM EDTA in PBS, and either analyzed immediately using flow cytometry or used for degranulation assays.

### Degranulation assay and IFN-γ production

After 24 hours of incubation with entinostat or vehicle, NK cells and tumor cells were cocultured in a 1:1 ratio for 6 hours in NK cell media as described above. Intracellular staining for IFN-γ was completed as previously described [54] and analyzed along with extracellular CD107a using flow cytometry.

### Flow cytometry

Flow cytometry analyses were conducted in LSR-II (BD Biosciences, San Jose, CA) or MACSQuant Analyzer 10 (Miltenyi Biotec, Bergisch Gladbach, Germany) and analyzed with FlowJo software (FlowJo LLC, Ashland, OR). Fixation/Permeabilization Solution Kit was used for intracellular staining according to the manufacturer’s protocol (BD Biosciences, San Jose, CA). The following antibodies were used in this study: DNAM-1 (11A8), NKp80 (5D12), CD155 (SKII.4), CD112 (TX31), CD107a (H4A3), CD3 (UCHT1) (BioLegend, San Diego, CA), Violet Fluorescent Reactive Dye, NKG2D (1D11), PD-1 (MIH4), HLA (W6/32), MICA/B (6D4), PD-L1 (MIH1), IFN-γ (4S. B3) (Invitrogen, Thermo-Fisher Scientific, Waltham, MA), NKG2A (REA110), KIR2DL1 (REA284), KIR2DL2 (DX27), KIR2DS4 (JJC11.6), KIR3DL1 (DX9), KIR2DL5 (UP-R1), CD56 (AF12-7H3) (Miltenyi Biotec, Bergisch Gladbach, Germany), NKp46 (195314), ULBP1 (170818), ULBP2,5,6 (165903), and ULBP3 (166510) (R&D Systems, Minneapolis, MN). Data were acquired using a MACSQuant Analyzer 10 (Miltenyi Biotec, Bergisch Gladbach, Germany) and analyzed using FlowJo software (Ashland, OR).

### RNA sequencing

Total RNA was extracted by Trizol from NK cells exposed to entinostat and DMSO for 24 hours. (*n* = 3 per group), followed by poly-A-purification, transcription, and chemical fragmentation using Illumina’s TruSeq RNA library kit (Illumina, Inc., San Diego, CA). Individual libraries were prepared for each sample, indexed for multiplexing, and then sequenced on an Illumina HiSeq2500. Transcript fragments were aligned to GRCh37 and quantified using Salmon [55]. Libraries were normalized, and differential gene expression analysis was conducted using DESeq2 [56]. Log fold-change shrinkage was completed using *apeglm* [57]. Fragments per kilobase million (FPKM) were calculated using the Fragments Per Kilobase of transcript per Million (FPKM) mapped reads. In RNA-Seq function in DESeq2 [56]. Gene set enrichment analysis was performed using Fast Gene Set Enrichment Analysis [58] using curated pathways from the Molecular Signatures Database (MSigDB) [38, 59–61]. Normalized enrichment score (NES) was calculated by using a mean enrichment score of random transcripts of the same size gene-set.

### Assay for Transposase Accessible Chromatin (ATAC) sequencing

DNA was extracted from the same samples that were utilized in the RNA-Seq experiment. Sample preparation was completed as previously described [62]. Sequence adapter trimming, alignment to the human genome, and post-alignment quality control were conducted using the ENCODE ATAC-seq analysis pipeline [63, 64]. Regions of chromatin accessibility were detected using three biological replicates of each treatment using Genrich [65]. Accessible regions of the genome that were only present within the treatment group were obtained by subtracting regions present within the control from the treatment group using BEDTools [66]. Accessibility peaks were annotated using Homer [67]. These annotated accessibility peaks were filtered to only include a protein-coding feature and feature less than 1000 bp from a transcription start site. Visualization of peaks utilized Integrative Genomics Viewer [68].

### Western blot

NK cells were isolated from three normal human donor buffy coats and exposed to entinostat and DMSO for 24 hours. Whole NK cells were lysed using RIPA buffer (20 mmol/L Tris HCl, pH 8.0, 150 mmol/L NaCl, 1 mmol/L disodium EDTA, 1 mmol/L EGTA, 2.5 mmol/L sodium pyrophosphate, 1 mmol/L β-glycerophosphate, 1% triton-X100) plus Halt protease/phosphatase inhibitor cocktail (ThermoFisher Scientific). Protein concentration was measured by BCA assay (ThermoFisher Scientific). Twenty micrograms of protein were run on 4% to 15% SDS-PAGE Precast TGX gels (Bio-Rad) and transferred to PVDF membranes (Bio-Rad, Hercules, CA). The membrane was blocked in TBS (pH 7.4) plus 0.1% Tween-20 and 5% bovine serum albumin, incubated overnight at 4°C with 1:2000 diluted antibodies. Antibodies used included TMEM173/STING (D2P2F), STAT4 (C46B10), pSTAT4 (D2E4) (Cell Signaling Technology, Danvers, MA), and IFIT1 (polyclonal) (Invitrogen, Thermo-Fisher Scientific, Waltham, MA). Membranes were incubated with goat anti-rabbit IgG (H+L)-HRP conjugated antibodies, washed with TBST, and developed with SuperSignal West Dura Extended Duration reagent (ThermoFischer Scientific) for chemiluminescence detection. Resulting bands were quantified and normalized to total protein using ImageJ software.

### Statistical analysis

Effect of entinostat on NK cells and tumor alone was analyzed using a ratio paired *t-test*. Degranulation and IFN-γ production experiments were analyzed using one-way ANOVA with Geisser-Greenhouse correction of the four different experimental comparisons. Post hoc analysis of each pair of experimental comparison was completed using the paired *t-test*. All statistical analysis was completed using GraphPad Prism 7 (San Diego, CA).

## SUPPLEMENTARY MATERIALS


