# Supplementary material for: Entinostat augments NK cell functions via epigenetic upregulation of IFIT1-STING-STAT4 pathway
Source: Oncotarget. 2020 May 19;11(20):1799–815. doi: 10.18632/oncotarget.27546 (PMC7244011; doi:10.18632/oncotarget.27546)
Supplement: Supplementary file 1 [file oncotarget-11-1799-s001.pdf]

Entinostat augments NK cell functions via epigenetic upregulation of IFIT1-STING-STAT4 pathway

SUPPLEMENTARY MATERIALS

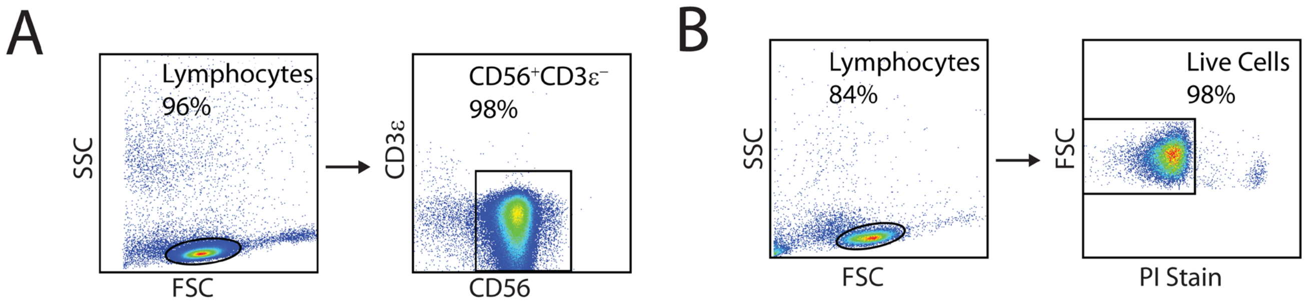

Supplementary Figure 1: Effect of entinostat on viability. (**A**) Flow cytometry scatter plot showing methods and purity of the isolated human NK cells from PBMC. (**B**) NK cell viability is not affected by the treatment of entinostat. Flow cytometry scatter plot showing the percentage of live lymphocytes after 24 hours incubation in entinostat.

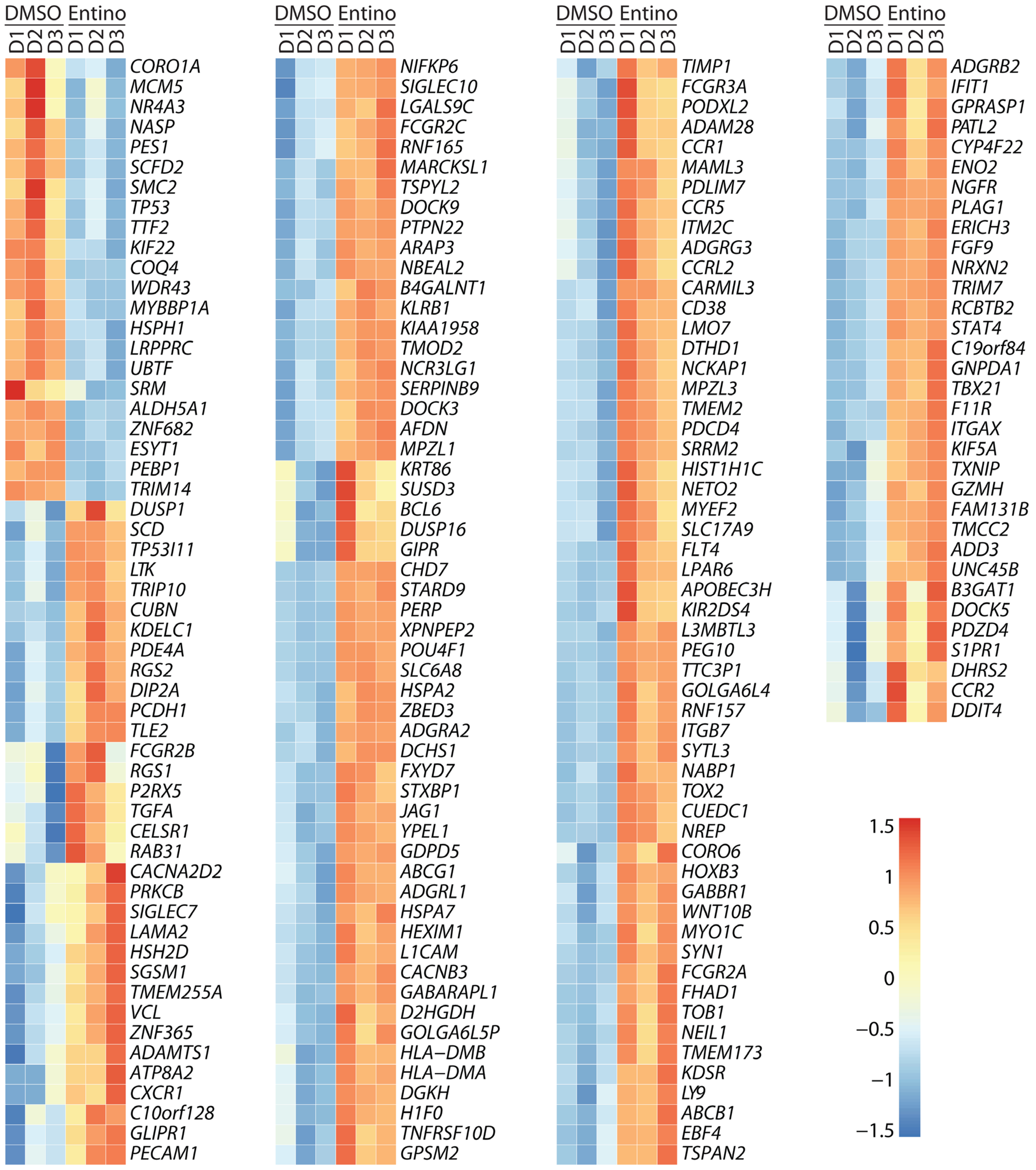

Supplementary Figure 2: Differentially expressed genes following entinostat treatment. Heatmap displaying the bottom 20 and top 200 genes in transcriptome by FDR < 0.05 in RNAseq data. Data shown are obtained by treating purified NK cells from three healthy donors per group with entinostat or DMSO.

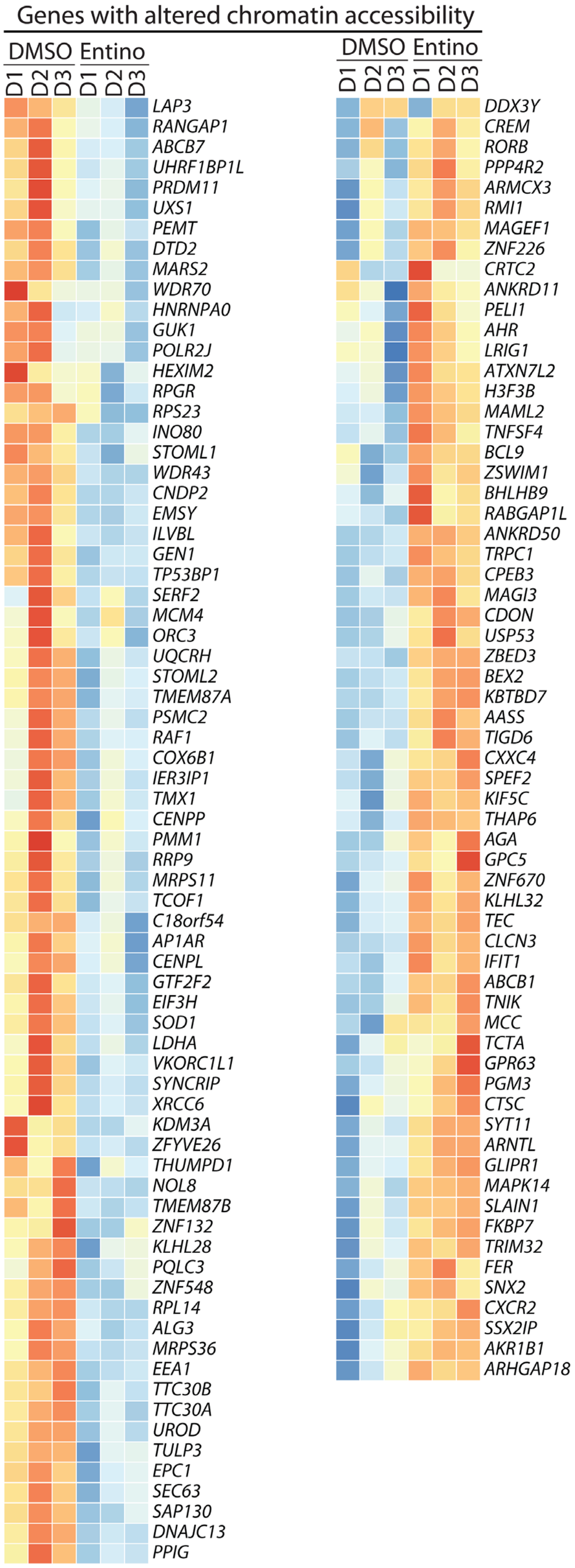

Supplementary Figure 3: Full set of genes identified by ATAC-Seq. Gene-expression heatmap displaying 135 genes with significant peaks identified via ATAC-Seq and with FDR < 0.05 in RNA-Seq analysis. Regions of chromatin accessibility were detected using three independent biological replicates (three healthy donors) of each treatment using Genrich (Github, https://github.com/jsh58/Genrich). Data shown are obtained by treating purified NK cells from three healthy donors per group with entinostat or DMSO.
